# Publication Trends in Drug Delivery and Magnetic Nanoparticles

**DOI:** 10.1186/s11671-019-2994-y

**Published:** 2019-05-16

**Authors:** Saba Ale Ebrahim, Amirhossein Ashtari, Maysam Zamani Pedram, Nader Ale Ebrahim

**Affiliations:** 1 Faculty of Electrical Engineering, K.N. Toosi University of Technology, Tehran, Iran; 2 Centre for Research Services, Institute of Management and Research Services (IPPP), University of Malaya (UM), Kuala Lumpur, Malaysia; 3 RVnIC, Iranian Center for Development Studies (ICDS), Tehran, Iran

**Keywords:** Magnetic nanoparticles, Biomedical and medical applications, Drug delivery to the brain/cell, Nanotechnology, Bibliometrics, Research productivity

## Abstract

This bibliometric study investigated the public trends in the fields of nanoparticles which is limited to drug delivery and magnetic nanoparticles’ literature published from 1980 to October 2017. The data were collected from the Web of Science Core Collections, and a network analysis of research outputs was carried out to analyse the research trends in the nanoparticles literature. Nanoparticles and its applications are progressing in recent years. The results show that documents in the field of nanoparticles in chemistry and material science have improved in citation rate, as the authors were researching in multidisciplinary zones. Top-cited documents are mainly focusing on drug delivery, magnetic nanoparticles and iron oxide nanoparticles which are also the top research keywords in all papers published. Top-cited papers are mostly published in Biomaterials journal which so far has published 12% of top-cited articles. Although research areas such as contrast agents, quantum dots, and nanocrystals are not considered as the top-ranked keywords in all documents, these keywords received noticeable citations. The trends of publications on drug delivery and magnetic nanoparticles give a general view on future research and identify potential opportunities and challenges.

## Introduction

Nowadays, nanoscale structures are widely proposed and attracted many researchers’ attention for usage in cellular biology [1]. The significant advances in nanotechnology are the reason for this attraction. Between various issues in the pharmacological field, developing beneficial drug delivery systems is one of the important key factors [2]. The main concern is focused on improving drug delivery efficiencies which are generally described in low disruptions, sustainability, and accurate and precise targeted delivery control [3].

In the past few decades, drug delivery systems based on the usage of magnetic behaviour of magnetic nanoparticles have been studied, and several types of research have been accomplished in this field [4–7]. Regarding the recent studies in drug delivery system, many methods have been proposed. Carbon-based nanotubes (CNTs) are a new method of drug translocating into targeted places inside the human body which are functionalised with proteins and peptides. Concerning low toxicity and high biocompatibility of functionalised CNTs, they are widely used in many nanobiotechnology application [3]. Beside the nanocarriers method, some researchers used bare nanoparticles as a novel method for brain disorder detection. They have crossed bare nanoparticle through the blood-brain barrier [8–10] towards the brain, and regarding the magnetic behaviour of the epileptic area, magnetic nanoparticles are aggregated in the defined area [11]. This bibliometric study investigated the public trends in the fields of nanoparticles, which is limited to drug delivery and magnetic nanoparticles’ literature.

Bibliometrics refers to the implementation of statistical methods for evaluating the research productivity, for individuals, institutes, and countries [12]. Bibliometrics is measuring academic performance based on various indices such as the number of publications, number of citations, and average citation per year [13]. The results of the bibliometric analysis can shed light on the factors that strengthen the contribution of studies in a research area and guide scholars towards producing impactful studies [14].

Bibliometric study analysis research productivity [15], top-cited publications [16], countries’ scholarly outputs [17], assessment of scientific activity [18], keywords selections effect on citations [19], effect of social media on research impact [20–22], international collaborations [23, 24], and increasing visibility and enhancing impact of research [25, 26], and compares the relative scientific contributions of specific research area, groups, or institutions [27]. Top-cited or highly cited papers are defined as those papers that received the highest number of citations in a certain period [28]. There has been an emerging interest in using top-cited papers as indicators in research assessments during the last decade [29]. The limited bibliometric study has been investigated on the publication patterns in nanoparticles, specially “magnetic nanoparticles” and “drug delivery”. A search on the Web of Science database for all bibliometric publications in the field of nanoparticles reveal seven documents [30–36]. Only one study [30] evaluated the scientific literature on drug delivery in the fields of nanoparticles which the study limited to 1974–2015. So, a comprehensive and up-to-date bibliometric study on “nanoparticles” is needed. This paper reports on the use of a bibliometric approach to analyse the productivity and development of publications on the title of nanoparticles in the period 1980–2017.

Web-based citation databases such as Scopus and Web of Science (WoS) are frequently used for deriving bibliometric data [37]. Since WoS is the oldest citation database, it has strong coverage with bibliometric data which goes back to 1900 [38]. The Web of Sciences Core Collection (as a part of WoS) is a leading database with high-quality and multidisciplinary research information, by the subscribed from the Institute of Scientific Information (ISI), also known as Thomson Reuters [13].

Bibliometrics cannot be a substitute for qualitative peer evaluation. Therefore, it should be used with precautions to evaluate the scholarly outputs [39]. So, qualitative analysis beside bibliometric study will elaborate more insight into scholarly outputs [40]. Therefore, in this study, the growing trend of documents published in recent years in the field of nanoparticles is considered. The Web of Science database was used to make a bibliometric analysis of nanoparticles research references during the years 1980–2017. However, the first article on drug delivery and magnetic nanoparticles was published in the year 2003. The main goal of this paper is to identify and analyse the top-cited papers researching on the field of nanoparticles to find a pathway for future research. The quantitative and qualitative analysis of the top-cited papers on drug delivery and magnetic nanoparticles gives a general view on current research and guideline for future research. Variant bar charts were plotted based on terms such as publication year, author, publication, keyword, and country to provide additional insights. The goal is to demonstrate the research status of the nanoparticles research field during this recent period.

## Methodology

The data were collected from the Web of Science Core Collection database on 17 October 2017. All Web of Science Core Collection citation indexes including Science Citation Index Expanded, Social Sciences Citation Index, Arts & Humanities Citation Index, Emerging Sources Citation Index, and relevant conference proceedings citation index were searched for “Nanoparticle*” in the title of documents. The results refined by “Magnetic Nanoparticle*”, and “Drug delivery” in the topic of documents. The results consisting 2066 documents, which includes all bibliometric data during the interval between the year of publication and 17 October 2017, were collected. To compare the differences between data collection from SCOPUS and WoS databases, the researchers run (TITLE (“Nanoparticle*”)) AND (TITLE-ABS-KEY (“Magnetic Nanoparticle*” AND “Drug delivery”)) search on SCOPUS database and found 1368 documents. Therefore, the WoS database is more comprehensive and the final analysis is carried out on WoS data sets.

After collecting the final data, a networking visualisation software called VoSViewer (http://www.vosviewer.com/) was used to demonstrate the publication output based on each grouping colour code. The abbreviation “VOS” in the VOSViewer stands for “visualisation of similarities” [41]. VOSviewer is a computer programme that plotted a relevance distance-based map and clustered keywords from the text in titles and abstracts of documents [42]. There are many softwares for mapping and visualisation, such as BibTechMon, Bibexcel, CiteSpaceII, CoPalRed, IN-SPIRE, Leydesdorff’s Software, Network Workbench Tool, Sci2 Tool, Vantage Point, and VOS Viewer [43]. The VOSviewer is one of them that is dedicated for bibliometric maps, scientific research, and graphical representation.

In order to analyse scholarly outputs in the area of the research, a web-based software called HAMMER was used. HAMMER is a web-based server for automating a network analysis for literature study scripts [44]. In the quality analysis of the documents, the top 100 documents with the most citation per year were investigated. There were 42 research papers and 57 review papers on the top of highly cited ratio documents. In this study, the 42 research articles were analysed qualitatively.

To map the present subtopics of the nanoparticle-based research field, especially drug delivery and magnetic nanoparticles, data tables are drawn to identify all 42 references in 2 dimensions. At first, the subjects focused on these studies are surveyed individually and the second dimension is the research methods applied in these documents. The data is produced by focusing on the article text, especially the abstract section. The outcomes of this set of top-cited references identify openings for future research.

## Result and Discussion

### Analysis of Publication Years

**Fig. 1:**
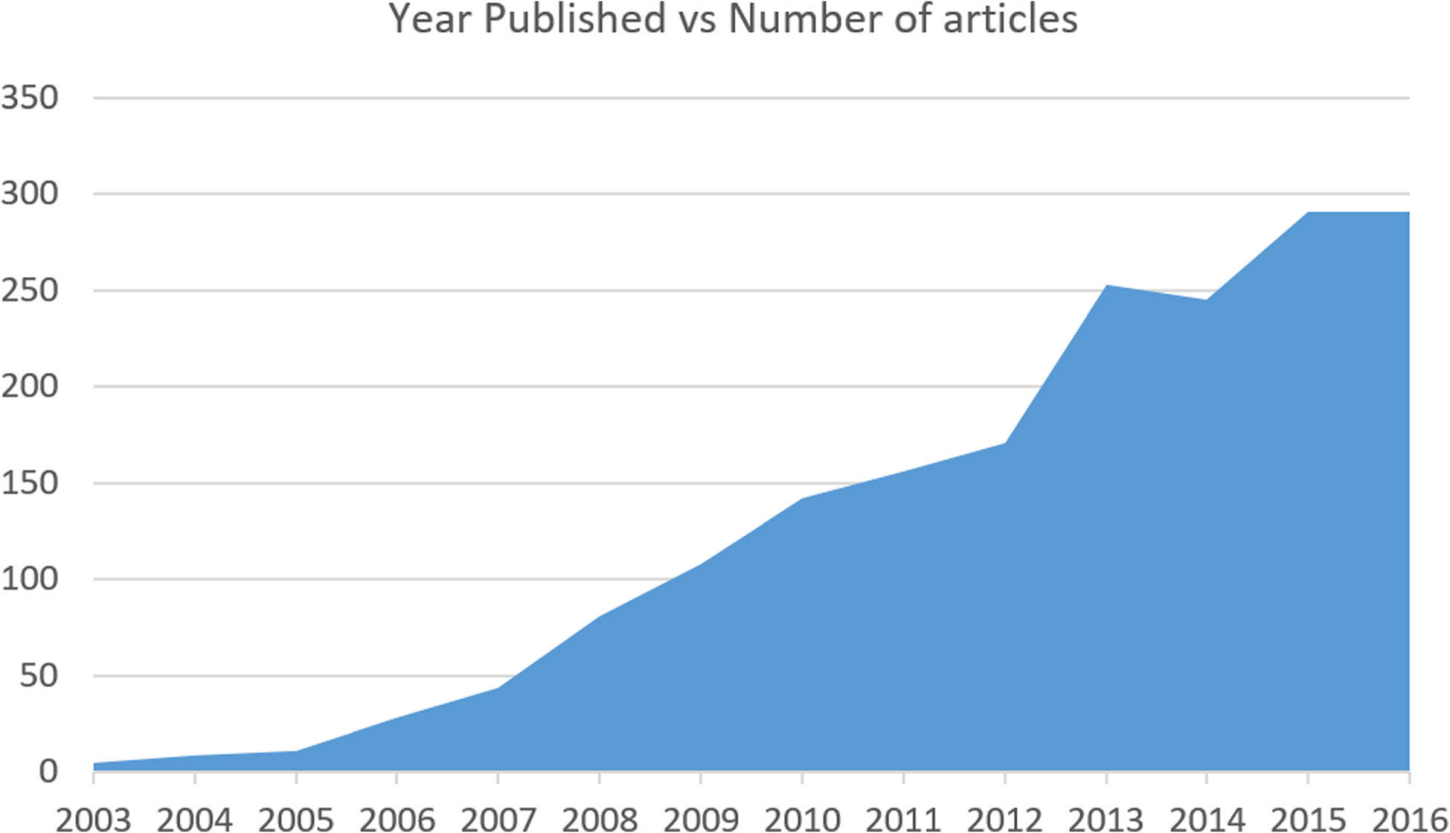
Publication years sorted by the number of articles published

Figure 1 shows the dispensation of published articles from the year 2003 to the first half of 2017. There are a different number of publications in variant sections. As it is shown in Fig. 1, in 2003–2012 period, the number of publications grows gradually with an upward curve trend from five articles published in 2003 to about 171 articles in 2012. There is a prompt rise around 2013 reaching to approximately 253 publications in that year. We can notice a rare decrease in 2014 coming down from 253 to 245. But after that, in 2015, the reduction has been compensated and the number of articles published has reached to nearly 291. This number of articles is 14% of all articles published in all times.

### Analysis of Authors

**Fig. 2:**
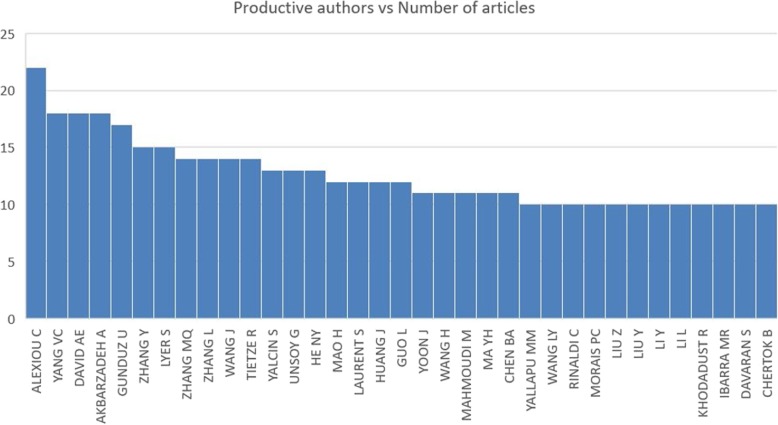
Important authors with the number of articles published

**Fig. 3:**
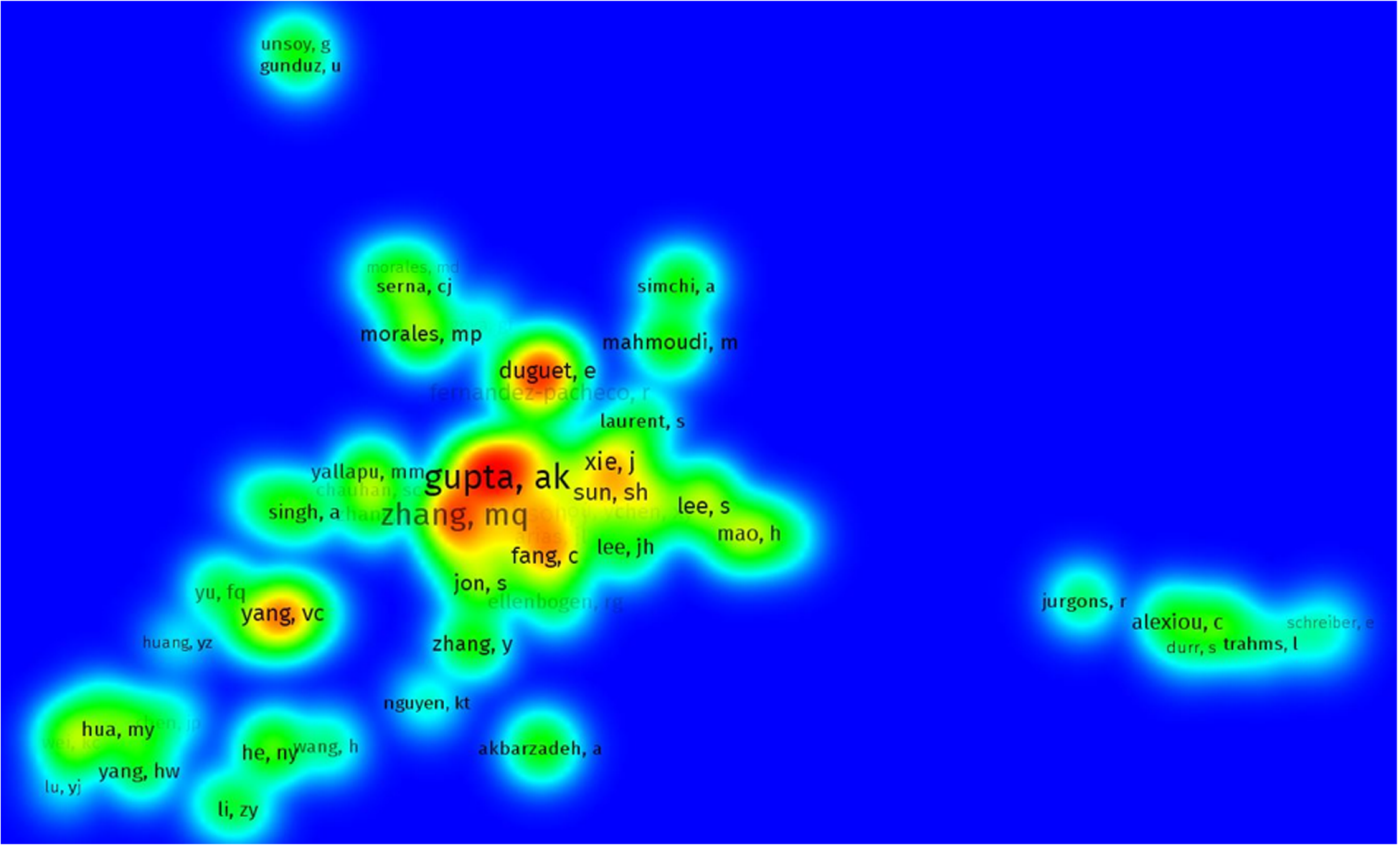
Important authors with the number of article citations

As Fig. 2 shows, the most active author is Alexiou C who has taken part in over 22 articles out of the overall number of 2066 in the field of nanoparticles. This huge number of publication equals to above 1% of publications at all times. Table 1 has the first 10 authors listed with their article count. Authors such as Yang VC, David AE, and Akbarzadeh A have participated in as high as 18 articles and Gunduz U in 17 publications relating to drug delivery and nanoparticles. They appear on the second to the fifth row of Table 1. According to our research, more productive authors such as Zhang Y and Lyer S are owing as far as 15 articles published about nanoparticles. Figure 3 illustrates the most cited author in the world, AK Gupta, that has the highest article citations with a great difference. His papers are generally about the narrow size of particles which leads them to their fantastic uniform physical and chemical characteristics [45] and the way they are nowadays used for variant biomedical applications [46]. As we can see in the figure Zhang MQ, Duguet E, Yang VC, and Jin Xie have earned the most citations after Prof. AK Gupta prospectively.

**Table 1:** Top 10 authors with their number of published articles

Authors	Number of articles published
Alexiou C	22
Yang VC	18
David AE	18
Akbarzadeh A	18
Gunduz U	17
Zhang Y	15
Lyer S	15
Zhang MQ	14
Zhang L	14
Wang J	14

### Analysis of Publications

**Fig. 4:**
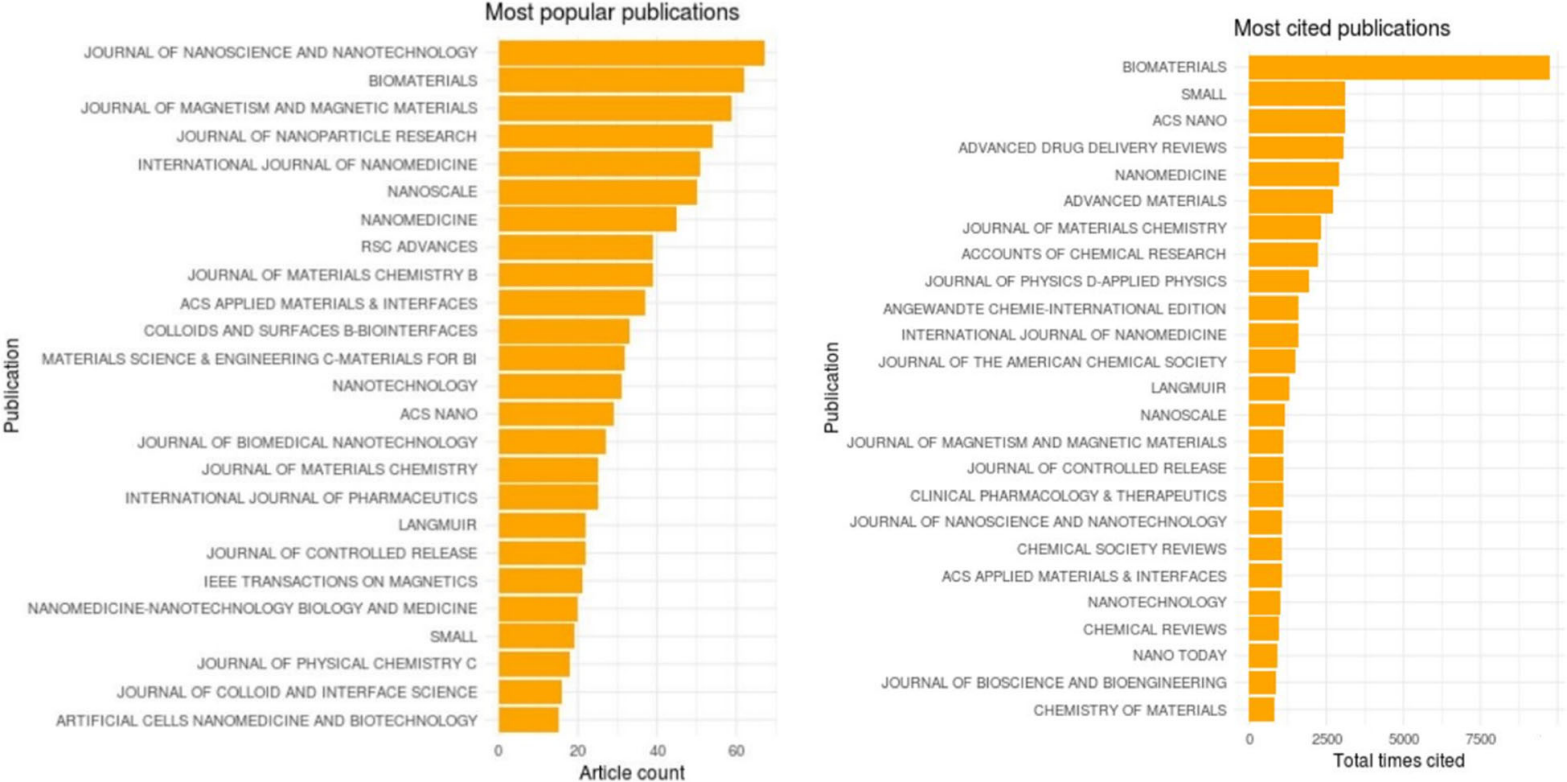
Important publications with the number of articles in their dataset and their citations

In Fig. 4, the Journal of Nanoscience and Nanotechnology [16] with over 65 articles in the field of nanoparticles has the most articles published. Nearly 62 articles have been published in Biomaterials. This journal has the most cited publications with 10,000 total times cited. Journal of Magnetism and Magnetic Materials from the Netherlands holds third place for the most popular publications with approximately 60 articles. A German journal called Small and ACS Nano along with Advanced drug delivery reviews are at the top owing about 3000 citations.

### Analysis of Keywords

**Fig. 5:**
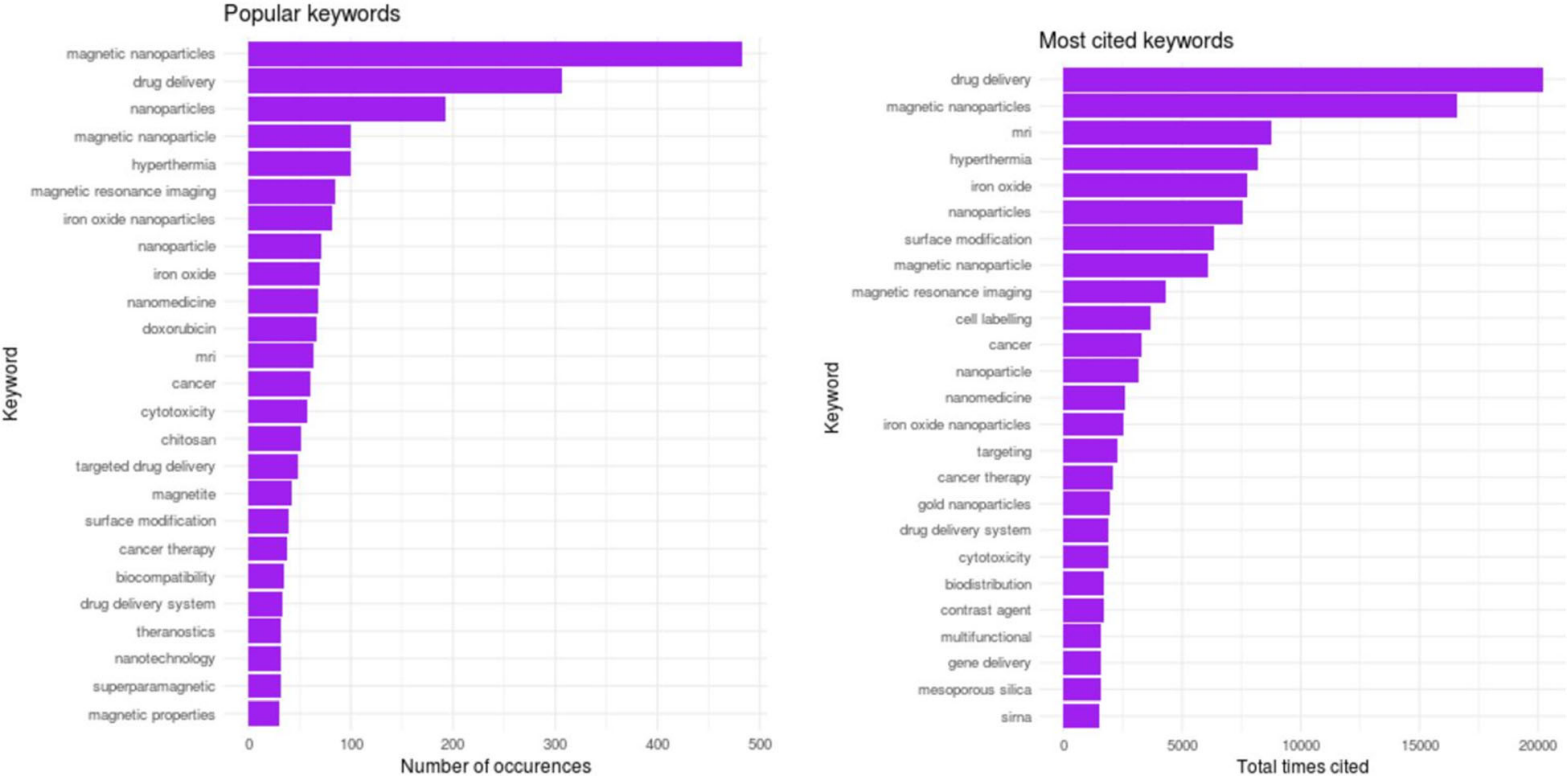
Important keywords mentioned and the number of their citations

Analysing different keywords assists researchers to explore dominant research topics. Top 10 keywords with the most citation are shown in Fig. 5. The word “Magnetic Nanoparticles” occurred more than 475 times with around 16,000 citations. The second common searched keywords are “Drug Delivery” which was used just above 300 and mentioned over 20,000 times. In summary, the most popular keywords in our research area, as Fig. 5 informs, are drug delivery, magnetic nanoparticles, MRI, hyperthermia, iron oxide, nanoparticles, surface modification, magnetic nanoparticle, magnetic resonance imaging, and cell labelling cited from 20,000 to 4000 times. Words such as siRNA, mesoporous silica, gene delivery, and multifunctional are the least cited ones with less than 2000 citations.

### Analysis of Countries

**Fig. 6:**
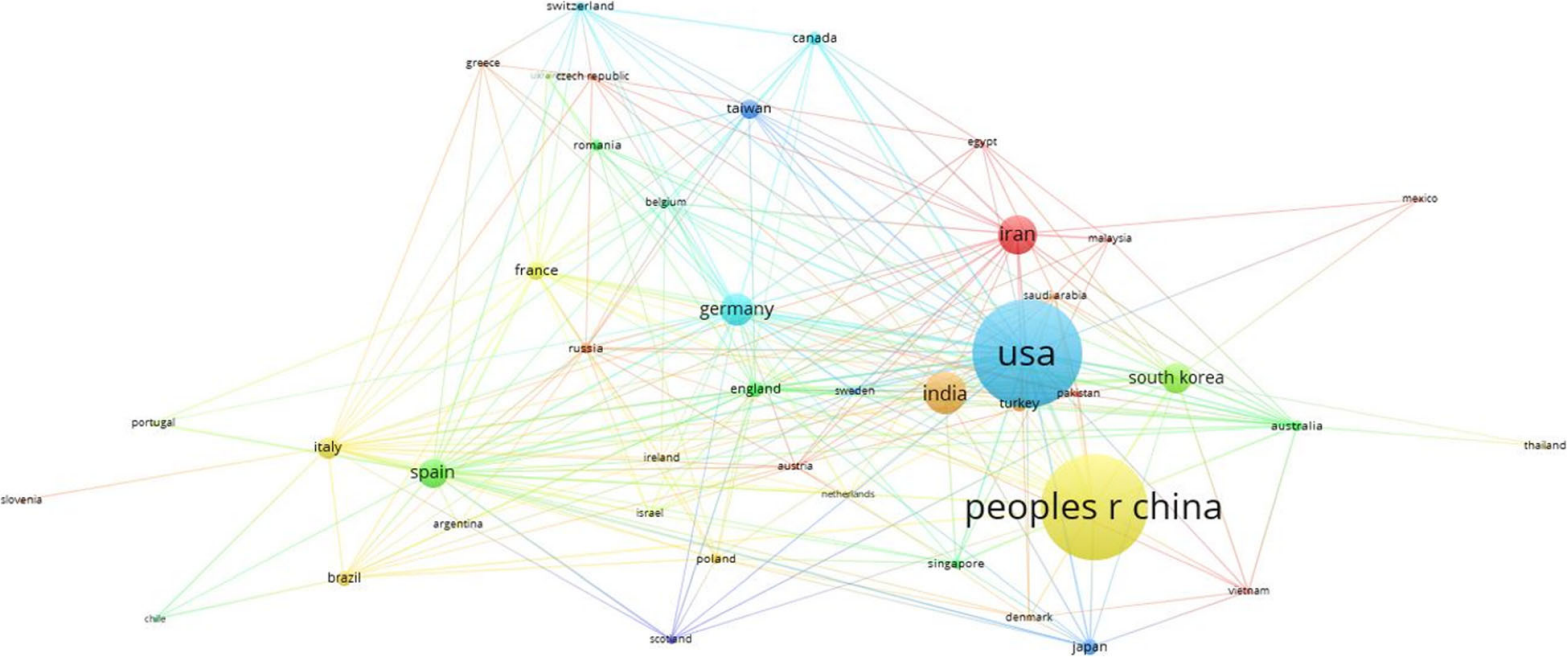
Countries sorted by the number of articles published

Figure 6 gives a complete picture of nanoparticle research all around the world. This repartition guide presents such important data for analysts to discover the place which they should start working on or building up some cooperation. Researches illustrate that 2066 papers were written in 73 countries. The top 10 countries in this field with their number of publications are shown in Table 2 as they represent 87.71% of all publications. The USA, China, India, and Iran have the greatest noteworthy number of publications compared to other nations. Japan, as a developed country, has not been focusing on this branch, but countries such as Italy, Taiwan, and France are shining in the eighth, ninth, and tenth rows, respectively. Germany has 123 published articles from the total number of 2066 papers in nanoparticles, this makes it ranked fifth. South Korea and Spain both have accounted for 5.3% of total publications, holding sixth and seventh place, respectively.

**Table 2:** Top 10 countries with their number of published articles

Countries	Number of articles published
USA	480
China	466
India	163
Iran	148
Germany	123
South Korea	111
Spain	110
Italy	78
Taiwan	70
France	63

#### Top 100 Cited Papers

There are 2066 documents analysed quantitatively in this study. By sorting them with their number of citations per year, it is understood that there are only 7 papers which have been cited over 100 times per year. Setting the threshold to 21 citations per year results in the top 100 papers. According to [47–50] papers, it is conventional to analyse the top 100 with the most citations per year. This analysis will emphasise the top 100 journals, top related keywords, top countries, and top sub-research areas in the field of nanoparticles. After that, the reader would be able to choose from the keywords and research areas before starting his/her research in order to aim for a great number of citations per year. The other benefit of this analysis is the idea of which journal is more suitable for submitting the nanoparticle-based article or review papers.

Most review papers gain a higher number of citations per year compared to the articles in the same field [20]. Review papers talk about the background of studies and give the reader a general idea of what he/she should do further in this area; this is one of the reasons for using the review paper more than articles which rises their citation number. The document types for the top 100 papers with the most citation/year ratio are 42% articles, 57 review papers, and only 1 review paper from a book chapter. In this study, we are going to analyse the data both in quantitative and qualitative ways based on the 100 top-cited papers and 42 top-cited articles, respectively.

### Quantitative Analysis

#### Analysis of Keywords in the Top 100 Cited Papers

**Fig. 7:**
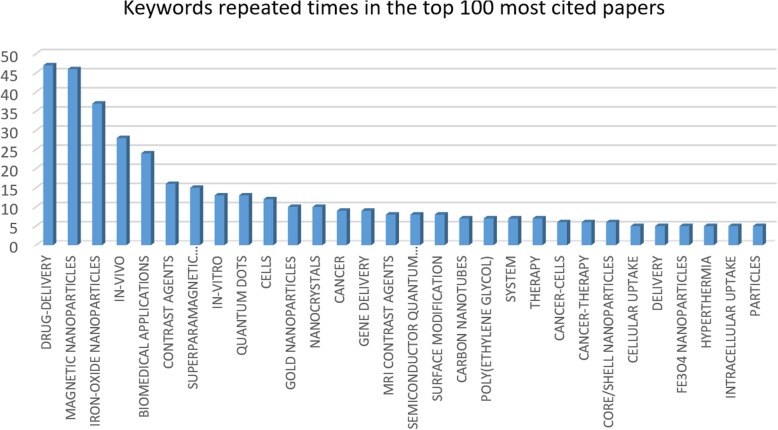
Keywords sorted by the number of articles published

One of the most important factors in a quantitative analysis is keywords analysis. There were 457 different keywords used in these top 100 papers. The graph in Fig. 7 shows keyword’s popularity, keywords repeated above 5 times, in the top 100 citation/year papers. As it is illustrated, the words Drug-Delivery and Magnetic Nanoparticles as keywords have the most popularity among 455 others, repeated 47 and 46 times, respectively. Iron oxide nanoparticles is by a big difference in the third place recurred 37 times in 100 papers.

Recently, utilizing magnetic nanoparticles has become the objective drug delivery technique [51]. Furthermore, iron oxide nanoparticles have superparamagnetic characteristics, and it is used for MRI monitoring targeting the brain tumour or breast cancer as a drug delivery vehicle [52–54]. In conclusion, these three keywords are intertwined and together on the top of the most used keywords’ list. In-VIVO and Biomedical Applications are included in a quite same number of papers, 28 and 24, respectively. Contrast agents, Superparamagnetic nanoparticles, In-VITRO, and Quantum Dots are the keywords with almost the same popularity from 16 to 13 papers among the 100 most cited papers.

Nowadays, health is the most important factor in our lives, and any issue relevant to health like methods of drug combination for a therapeutic effect or any disease treatment for living creatures based on nanoparticles is of interests [55]. Drug delivery holds the first place among keywords in all papers and in the top 100 citations/year papers. The keyword Magnetic Nanoparticles is in second place in both rankings (Table 3). The reason is the popularity of all sorts of the application via magnetic nanoparticles such as boosting MRI data and tissue engineering methods, modifying drug delivery along with cancer diagnosis [56]. Iron oxide nanoparticles are ranked third via their useful utilisation in medical and biomedical applications. IO nanoparticles as an eco-friendly and non-toxic material have superparamagnetic characteristics and biomedical applications helping the world these days [57, 58].

**Table 3:** Comparing keywords ranking in the top 100 best cited papers with keywords ranking in all papers

Keywords	Ranking in top 100	Ranking in all
Drug-delivery	1	1
Magnetic nanoparticles	2	2
Iron-oxide nanoparticles	3	3
In-vivo	4	5
Biomedical applications	5	4
Contrast agents	6	13
Superparamagnetic nanoparticles	7	12
In-vitro	8	6
Quantum dots	8	17
Cells	9	9
Gold nanoparticles	10	11
Nanocrystals	10	18
Cancer	11	10
Gene delivery	11	16

Keywords number four and five from the top 100 citations/year papers have switched places in the ranking in all papers’ column. This means that although biomedical applications are more useful in comparison with In-VIVO experimentations, top 100 cited papers have used the keyword In-VIVO more than Biomedical Applications. Since In-VIVO is more limited and it has its own biomedical applications in other words the word Biomedical Applications contains subtopics such as In-VIVO, the top 100 citations/year papers have focused on the particular field called In-VIVO more than Biomedical Applications.

One of the interesting points of this comparison is the keyword Contrast Agents. Particular contrast agents are advanced for MRI, sonography, or X-ray tests [59]. An ideal future medical imaging, as contrast agents’ applications, is necessary in order to achieve treatments without side effects [60]. So, nowadays contrast agents and their medical imaging application are more important and conventional researches and the papers related to them will be cited more than papers with the topic called Cancer. Superparamagnetic nanoparticles such as IO nanoparticles, which is ranked 3, are used in variant biomedical applications. This is a quite common research topic holding the seventh position in the 100 top citation papers’ ranking and the 12th position in all papers’ ranking

In-VIVO keyword’s ranking is followed by In-VITRO keyword in the ranking of total papers, but there are three topics in between In-VIVO and In-VITRO in the top 100 ranking list. Overall, the benefit of In-VIVO experiments compared to IN-VITRO is that researchers are able to spot the effects on a living objective in its natural home and the upcoming results will be accurate [61]. This is the reason why nowadays In-VIVO researches are one of the highly cited researches.

#### Analysis of Research Area in the Top 100 Most Cited Papers

**Fig. 8:**
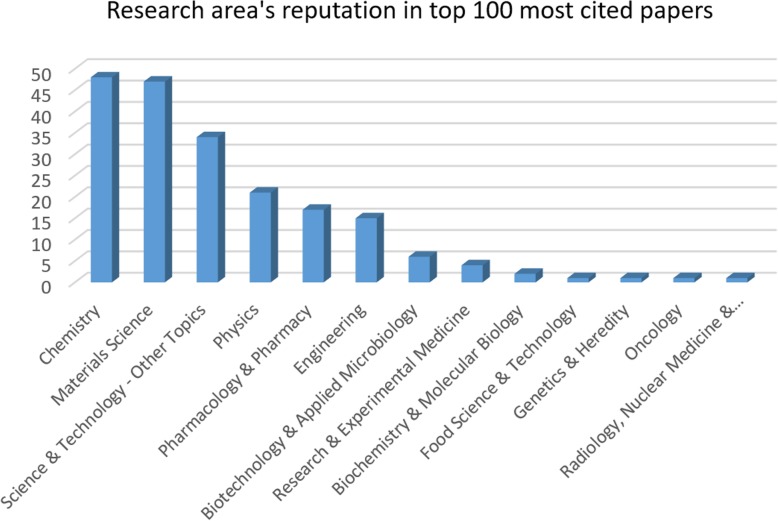
Research areas sorted by the number of articles published

In this analysis, we are going to analyse the data based on 100 top-cited papers and compare the rankings of research areas in 100 top-cited papers with research areas in all papers. There are respectively 13 and 37 different research topics used in top 100 best cited papers and in all papers. The graph in Fig. 8 shows the research area’s popularity, research area’s reputation, in the top 100 citations/year papers. In 100 papers including 42 articles and 57 review papers and a review on a book chapter, the most common research area is “Chemistry”. “Material Science” is in fact the second favourite research area with slight difference of only one paper.

Nanoparticles have variant applications in chemistry, for example, they are used as catalyst to enhance chemical reactions, as industrial water pollutant remover through chemical reactions, etc. [62]. So, these different researches have made chemistry number 1 of all, and 48 top-cited papers [out of 100] related to nanoparticles have been researched in chemistry. It is analysed that authors of the chemistry papers are not just experts in the single field of chemistry, but also they are different authors skilled in different categories such as biomedical engineering, molecular genetics and microbiology, physics, and radial diagnostics. The same pattern exists for material science research topic. Surprisingly, only few of the authors, as professors, were in the department of material science. For writing these sort of papers in the field of material science, it is necessary to gather authors with variant expertise in some of the field of medical physics, biological sciences, physics, chemistry and biochemistry, radiology, pharmacy, etc.

Science and technology–other topics are researched by 34 papers out of 100. By analysing the authors’ address, it is found that the phrase “other topics” in this research area mostly means areas such as bioengineering, biomedical engineering, and microelectronic. Physics, pharmacology and pharmacy, and engineering are the subjects which have been used 21, 17, and 15 times in 100 most cited papers, respectively. As it is shown in Fig. 8, chemistry, material science, and science and technology seems to be really involved with nanoparticles. Recently, they are much more controversial topics compared to physics, pharmacy, or engineering.

As it was discussed above, the key goal for the top 100 papers in order to get a high citation is researching in multidisciplinary zones and not just in pure physics, pharmacy, or even engineering alone. Table 4 compares research area’s rankings in 100 top-cited papers and the ranking in all papers. Overall, research topics such as chemistry and material science are at the same level, and somehow, they will switch places in the ranking list. The other areas for example physics, pharmacology and pharmacy, engineering and biotechnology and applied microbiology are ranked the same in both analyses.

**Table 4:** Comparing research areas repeated times in the top 100 best cited papers with research areas in all papers

Research area	Ranking in top 100	Ranking in all
Chemistry	1	2
Materials science	2	1
Science and technology–other topics	3	3
Physics	4	4
Pharmacology and pharmacy	5	5
Engineering	6	6
Biotechnology and applied microbiology	7	7
Research and experimental medicine	8	9
Biochemistry and molecular biology	9	8
Food science and technology	10	18
Genetics and heredity	10	17
Oncology	10	12
Radiology, nuclear medicine, and medical imaging	10	14

#### Analysis of Journals in the Top 100 Most Cited Papers

**Fig. 9:**
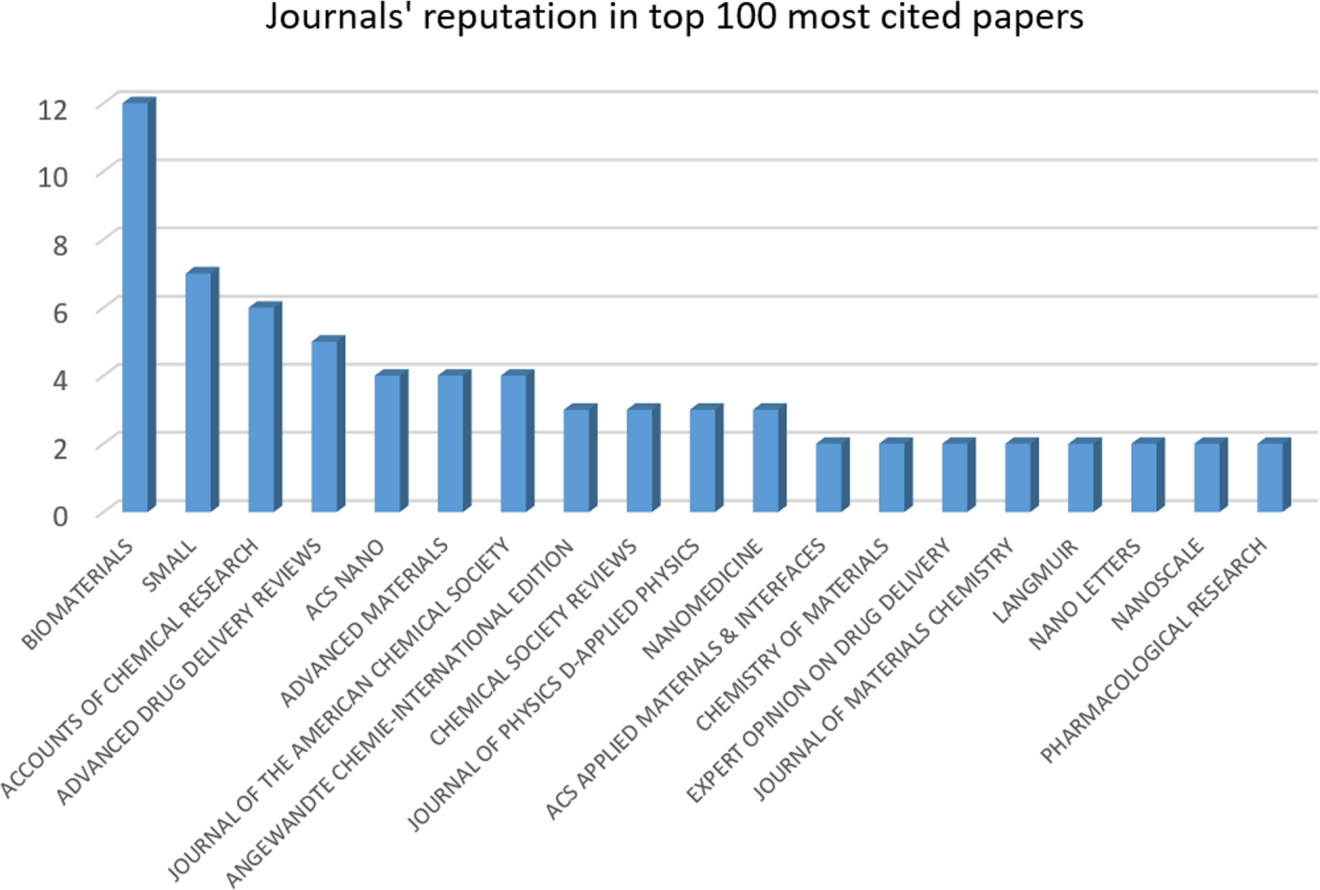
Journals sorted by the number of papers published (journals are used least two times)

Journals’ reputation in the top 100 most cited papers is shown in Fig. 9. The bar chart illustrates famous journals which have published at least 2 papers from the top 100 most cited papers in the field of nanoparticles. The journal of Biomaterials has, by far difference, the most papers published in comparison with other journals. It is obvious that this journal is more appropriate for a related paper to get a high citation per year rate. The journal of Small is the second most popular journal in this study, which has published 7 papers from the top 100 cited list. The journal of Accounts of Chemical Research along with the journal of Advanced drug delivery reviews is at the next places with the lower number of papers published in this area.

At this stage, the abovementioned journals are compared with journals which published the most papers in all nanoparticle-based references. It can be seen in Table 5 that the journal of Biomaterials remains the best, and it is considered as the second most articles published journal in the area of nanoparticles. Surprisingly, other journals are ranked completely different. Generally, the journals with the most cited papers in the field of nanoparticles are not the top 10 mostly used journals in all documents. So, this issue must be considered when finding an appropriate journal to submit in the field of nanoparticles, drug delivery, or magnetic nanoparticles.

**Table 5:** Comparing top 100 best cited papers’ journals with all journals

Journals	Ranking in top 100	Ranking in all
Biomaterials	1	2
Small	2	18
Accounts of Chemical Research	3	29
Advanced Drug Delivery Reviews	4	29
ACS Nano	5	13
Advanced Materials	5	26
Journal of the American Chemical Society	5	25
Angewandte Chemie-International Edition	6	27
Chemical Society Reviews	6	32
Journal of Physics D-Applied Physics	6	26
Nanomedicine	6	7
ACS Applied Materials & Interfaces	7	9
Chemistry of Materials	7	28
Expert Opinion on Drug Delivery	7	30

### Qualitative Analysis

In this study, we are going to analyse the data in a qualitative way based on 42 top-cited articles. The idea is to know the important topics in nanoparticles which have been studied the most or the least used topics that have been making progress (shown in Table 6).

**Table 6:**
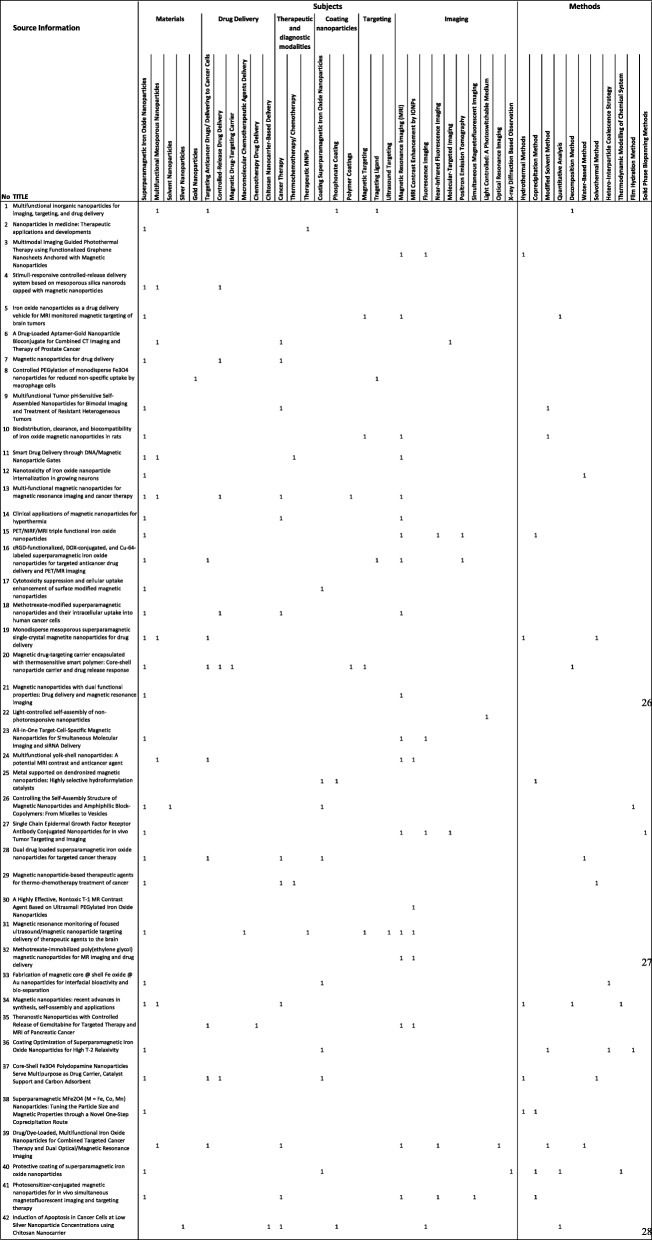
Summary of the top 42 most cited articles in the field of study

#### Subjects of Nanoparticles Studies

This analysis endeavours to discuss materials, drug delivery, therapeutic and diagnostic, coating, targeting, and imaging perspectives of nanoparticles’ studies. The most used topics and their subtopics are considered in this study. Most articles in the top 42 most cited articles’ list, in our data set, focus on superparamagnetic iron oxide nanoparticles. This material is commonly used as nanoparticles with magnetic properties which is also called magnetic nanoparticles, superparamagnetic nanoparticles, or iron oxide nanoparticles. Half of the magnetic nanoparticle-based articles have been using magnetic resonance imaging as their imaging process. There is only a single article using therapeutic MNPs [63].

Multifunctional mesoporous nanoparticles are the second most popular materials which sometimes overlaps with the magnetic nanoparticles in some references [64–68]. Silver is used as a nanoparticle material only once in the 42 most cited articles. The silver nanoparticle-based article has its unique way of drug delivery, a chitosan nanocarrier (NC)-based delivery using fluorescence imaging. It has been used in cancer therapy along with magnetic nanoparticles and multifunctional mesoporous nanoparticles [69].

Drug delivery is a popular study among nanoparticle studies. Subtopics such as targeting anticancer drugs, delivering drugs to cancer cells, or controlled-release drug delivery has allocated over a quarter of our database articles’ subjects. It is found that as rare as one research is based on a thermotherapy or chemotherapy of cancer [70] and other cancer therapy articles have researched on the help of controlled-released drug delivery or magnetic resonance imaging or even both on cancer therapy. Fluorescence imaging and near-infrared fluorescence imaging methods are used in articles published from 2009 to 2011. This is a proof that the method has had its progress and its citation per year rating is downgrading in recent years.

Among all different research and experiments on coated nanoparticles, coating superparamagnetic iron oxide nanoparticles has become famous. It is interested that none of the coating superparamagnetic iron oxides have been using MRI imaging unlike other types.

#### Methods of Nanoparticles Studies

The analysis of variant methods used by 42 top-cited articles shows the use of either common or particular different methods of nanoparticle-based studies. Each popular method has been utilised in 5 to as low as 1 article among all 42 top-cited articles. Methods such as hydrothermal methods, coprecipitational method, modified solvent method, quantitative analysis, decomposition method, water-based method, solvothermal method, hetero-interparticle coalescence strategy, thermodynamic modelling, film hydration method, and solid-phase biopanning methods are used in this set of papers.

Hydrothermal methods are quite popular using superparamagnetic iron oxide nanoparticles. Solvothermal methods are considered hydrothermal where the solvent is water. There are researches done by both hydrothermal and solvothermal methods based on superparamagnetic iron oxide nanoparticles [67, 71]. Modified solvent methods along with solvothermal methods are applied in recent experiments [70–73].

In this study, 42 references with the highest citation/year rate on nanoparticles were reviewed. Most of the references have nominated superparamagnetic iron oxide nanoparticles as the nanoparticles’ materials, followed by a few references focusing on targeting anticancer drugs or drug delivery for cancer therapy. A certain number of articles have been using magnetic resonance imaging, following a few considered fluorescence imaging, near-infrared fluorescence imaging, molecular-targeted imaging, and positron emission tomography as their imaging agents. In general, references’ arrangement has a connection with a wide scope of research goals. Nevertheless, the techniques used for nanoparticle-based researches are just divided into a few.

The limitation of this study is collecting data from the Web of Science Core Collection based on title search for “Nanoparticle*” with “Magnetic Nanoparticle*”, and “Drug delivery” in the topic. Therefore, documents in other databases such as SCOPUS were not considered. Although, the number of documents in the WoS database is higher than that in the SCOPUS database in this research area. There might be some relevant articles which talk about “Nanoparticle”, but the word “Nanoparticle” is not in the title of the paper. Such papers and also low-cited documents were not included in the quantitative and qualitative analysis. One of the merits of this study is to encourage the researchers to start their research in multidisciplinary zones and not just in pure physics, pharmacy, or even engineering alone. The 42 top-cited documents which were analysed qualitatively give an insight into the drug delivery and magnetic nanoparticles research area.

## Conclusions

In summary, an extensive bibliometric analysis of nanoparticles-based research documents was made with the help of the Web of Science database. Nanoparticle-based researches were characterised quantitatively and qualitatively from 2003 to 2017. The result shows an increase in the number of articles published during these years. Researchers from the USA and China contributed most of the publications. Analysis of keywords shows the stressed points in nanoparticle research field which guides to a direct and inform future. Chemistry and material science research areas are the most common areas using nanoparticles. The key factor for this success is researching in multidisciplinary zones and not just in pure physics or pharmacy or even engineering.

## Abbreviations

ISI: Institute of Scientific Information
ACS: American Chemical Society
CNTs: Carbon-based nanotubes
IO nanoparticles: Iron oxide nanoparticles
MNPs: Magnetic nanoparticles
MRI: Magnetic resonance imaging
NC: Nanocarrier
NSET: National Society for Earthquake Technology
siRNA: Small interfering RNA
VOS: Visualisation of similarities
WoS: Web of Science
